# Whole-Genome Saliva and Blood DNA Methylation Profiling in Individuals with a Respiratory Allergy

**DOI:** 10.1371/journal.pone.0151109

**Published:** 2016-03-21

**Authors:** Sabine A. S. Langie, Katarzyna Szarc vel Szic, Ken Declerck, Sophie Traen, Gudrun Koppen, Guy Van Camp, Greet Schoeters, Wim Vanden Berghe, Patrick De Boever

**Affiliations:** 1 Environmental Risk and Health unit, Flemish Institute for Technological Research (VITO), Mol, Belgium; 2 Laboratory of Protein Chemistry, Proteomics and Epigenetic Signaling (PPES), University of Antwerp, Wilrijk, Belgium; 3 Department of Biomedical Sciences, University of Antwerp, Wilrijk, Belgium; 4 Laboratory of Cancer Research and Clinical Oncology, Center for Medical Genetics, University of Antwerp, Edegem, Belgium; 5 University of Southern Denmark, Institute of Public Health, Department of Environmental Medicine, Odense, Denmark; 6 Centre for Environmental Sciences, Hasselt University, Diepenbeek, Belgium; Bellvitge Biomedical Research Institute (IDIBELL), SPAIN

## Abstract

The etiology of respiratory allergies (RA) can be partly explained by DNA methylation changes caused by adverse environmental and lifestyle factors experienced early in life. Longitudinal, prospective studies can aid in the unravelment of the epigenetic mechanisms involved in the disease development. High compliance rates can be expected in these studies when data is collected using non-invasive and convenient procedures. Saliva is an attractive biofluid to analyze changes in DNA methylation patterns. We investigated in a pilot study the differential methylation in saliva of RA (n = 5) compared to healthy controls (n = 5) using the Illumina Methylation 450K BeadChip platform. We evaluated the results against the results obtained in mononuclear blood cells from the same individuals. Differences in methylation patterns from saliva and mononuclear blood cells were clearly distinguishable (P_Adj_<0.001 and |Δβ|>0.2), though the methylation status of about 96% of the cg-sites was comparable between peripheral blood mononuclear cells and saliva. When comparing RA cases with healthy controls, the number of differentially methylated sites (DMS) in saliva and blood were 485 and 437 (P<0.05 and |Δβ|>0.1), respectively, of which 216 were in common. The methylation levels of these sites were significantly correlated between blood and saliva. The absolute levels of methylation in blood and saliva were confirmed for 3 selected DMS in the *PM20D1*, *STK32C*, and *FGFR2* genes using pyrosequencing analysis. The differential methylation could only be confirmed for DMS in *PM20D1* and *STK32C* genes in saliva. We show that saliva can be used for genome-wide methylation analysis and that it is possible to identify DMS when comparing RA cases and healthy controls. The results were replicated in blood cells of the same individuals and confirmed by pyrosequencing analysis. This study provides proof-of-concept for the applicability of saliva-based whole-genome methylation analysis in the field of respiratory allergy.

## Introduction

Respiratory allergies (RA) contribute significantly to the burden of chronic respiratory diseases worldwide. The World Health Organization estimated patients suffering from asthma at 235 million in 2013 [1] and the numbers for allergic rhinitis at about 400 million in 2006 [2]. Epigenetic processes and altered DNA methylation patterns in gene regulatory sequence regions are plausible pathways contributing to the development and progression of RA. Bégin and Nadeau recently reviewed the literature on epigenetic regulation of asthma and allergic disease. They noted that several loci, identified via candidate gene or genome-wide approaches, are associated with the disease phenotype and environmental exposures [3]. The research on environment-driven epidemic of RA has been fueled by the observation that early life events can have an impact on the prevalence of RA later in life [4–6]. Investigations making use of longitudinal cohorts are hampered because traditional blood sampling is an invasive approach—particularly in children and patient groups it is kept to a minimum for practical and ethical reasons.

Saliva has recently attracted a lot of attention because it contains a broad range of diagnostically relevant molecules (i.e. DNA, microRNA and antibodies). These biomolecules are useful to detect local mouth and throat diseases, but can also be used to predict/diagnose systemic diseases and health conditions [7]. For example, salivary cytokine profiles have been successfully used as biomarkers of respiratory and other immunological disorders in the field of clinical diagnostics [8–13]. In addition, it has been reported that it is a good source of high quality DNA for use in (epi)genomics [14–19]. Non-invasive saliva sampling improves compliance of individuals and allows multiple collections in one day without imposing too much discomfort. In addition, saliva is easy to collect, store and transport. Consequently, individuals can easily collect their saliva at home and then either transport it to their doctor or mail it to the research institute [20].

Several studies have identified epigenetic marks in blood to distinguish RA cases from healthy controls (e.g. [21–24]), while only 2 studies have so far reported the use of saliva for gene-targeted DNA methylation studies in the context of asthma [25,26]. A select set of studies focusing on healthy subjects have compared DNA methylation patterns in blood and saliva [14–16]. However, there are no studies available that used a case-control design to analyze and compare DNA methylation patterns in blood and saliva in individuals with RA. The aim of our study was to generate whole-genome DNA methylation profiles in saliva and compare those with the ones obtained from peripheral blood mononuclear cells (PBMC) from the same individuals. In addition, we investigated if differentially methylated sites could be identified when comparing individuals suffering from RA with healthy individuals. Illumina Infinium HumanMethylation450 BeadChips were used for genome-wide screening of differentially methylated sites (DMS) in PBMC and saliva in a pilot study of 10 volunteers. Verification of selected DMS was performed via bisulfite pyrosequencing.

## Materials and Methods

### Study design and sample collection

Non-smoking participants were recruited at the Flemish Institute for Technological Research (VITO) via personal contact or during their yearly routine medical follow-up. Ten adult volunteers, 5 females and 5 males, were recruited for this pilot study. Five participants suffered from RA and five individuals served as healthy controls. All volunteers completed a short survey (based on the International Study of Asthma and Allergies in Children (ISAAC) [27]) including questions on: A) occurrence and severity of (doctor diagnosed) RA symptoms (e.g. runny nose, sneezing, itchy eyes ever occurring and/or in the last 12 months), B) clinical management and medical treatment and C) family history of allergies (mother or father). An individual was classified as RA case, if at least one RA symptom occurred regularly, and was doctor diagnosed. The study was approved by the ethical committee of the University Hospital in Antwerp (file number 13/2/22, Belgian registration number B300201316329). Written informed consent was obtained prior to sample collection.

Blood samples (10 mL) were collected in EDTA tubes (BD Vacutainer®, BD, Plymouth, UK) and stored at room temperature (<2h) until further processing. Plasma was removed by centrifugation at 800xg for 5 min. Next, Lymphoprep™ (Axis-Shield, Oslo, Norway) was used to isolate a suspension of peripheral blood mononuclear cells (PBMC). The PBMC suspension was divided into two aliquots (each originating from 5 mL of the original whole blood sample) and spun down (10min, 300xg at 4°C) to create PBMC pellets, which were stored at -80°C till the DNA extraction. Unstimulated saliva samples (2 mL) were collected (within the first hour following the blood collection) using an Oragene DNA OG-500 self-collection kit (DNA Genotek, Ottawa, Canada). The saliva samples were kept at room temperature until DNA extraction.

### DNA extraction and bisulfite treatment

Genomic DNA (gDNA) was extracted from PBMC pellets using Gentra Puregene Blood Kit (Qiagen, Hilden, Germany) according to the manufacturer’s protocol. An RNase digestion step was included. gDNA was extracted from saliva using the Oragene PrepIT kit (DNA Genotek, Ottawa, Canada) according to the manufacturer’s instructions.

About 500 ng of gDNA was bisulfite converted using the EZ DNA methylation kit (Zymo Research, Cambridge Bioscience, Cambridge, UK) according to manufacturer’s instructions. Bisulfite conversion was obtained in a PCR cycling protocol (i.e. 16 x (95°C for 30 sec, 50°C for 60 min) and then hold at 4°C) that is recommended for methylation analysis on the Infinium HumanMethylation450 BeadChip Array (Illumina, San Diego, CA, USA). Successful bisulfite conversion was confirmed by the amplification of a 208 bp amplicon of the *SALL3* gene (95°C 15 min; then 45 cycles of 94°C 30 sec, 55°C for 30 sec, 72°C for 30 sec; followed by 72°C for 10 min) using the primer set: *SALL3*-Fw: 5'-GTTTGGGTTTGGTTTTTGTT-3'; *SALL3*-Rev: 5'-ACCCTTTACCAATCTCTTAACTTTC-3'. Additional internal sequence-specific bisulfite conversion controls were included in the pyrosequencing target gene assays.

### Infinium HumanMethylation450 BeadChip Array

Genome-wide DNA methylation profiles were generated with Infinium HumanMethylation450 BeadChip Array (Illumina, San Diego, CA, USA). 4 μl of bisulfite-converted DNA (~150 ng) was used for the whole genome amplification reaction, enzymatic fragmentation, precipitation and resuspension in hybridization buffer. Subsequent steps of DNA methylation analysis were carried out according to the standard Infinium HD Assay Methylation Protocol Guide (Part #15019519, Illumina). The BeadChip images were captured using the Illumina iScan. The raw methylation intensities for each probe were represented as methylation β-values (ranging from 0, unmethylated, to 1, fully methylated) and extracted from GenomeStudio Methylation Module software without background correction and normalization.

Data were analyzed using the RnBeads pipeline on the freely available statistical software platform R [28]. All samples passed quality controls and were used for further processing. Cg-probes were filtered out before normalization according to the following criteria: A) probes with a missing value (NA) in at least one sample, B) bad quality probes based on an iterative greedycut algorithm where a detection p-value of 0.01 is set as a threshold for an unreliable measurement, and C) probes containing more than two single-nucleotide polymorphisms of minor allele frequency (MAF) > 0.01. Methylation values of all remaining probes were within-array normalized using the Bèta Mixture Quantile (BMIQ) dilation [29]. After data normalization we implemented another filtering step which included removal of: D) probes measuring methylation in a non-CpG context, and E) probes on sex chromosomes. Hierarchical clustering was performed for studying the agreement of the methylation patterns between blood and saliva. We used average linkage and a correlation-based distance metric for clustering.

To estimate the proportion of various cell types in saliva and PBMC samples the statistical deconvolution method described by Houseman and colleagues was used [30,31]. Reference methylomes from leukocyte subtypes were obtained from the study of Reinius et al. [32] using the FlowSorted.Blood.450K R package. Buccal epithelial cells reference methylomes were obtained from the GEO dataset GSE46573 [33]. The reference methylation datasets were preprocessed in the same way as our dataset. The 100,000 most variable cg-probes were used to identify 500 probes associated with the cell types, and subsequently estimating the relative proportions of each cell type in our saliva and PBMC samples.

### Pyrosequencing

CpG site-targeted bisulfite pyrosequencing was used to confirm the Infinium HumanMethylation450 BeadChip Array results. Forward, biotinylated- reverse and sequencing primers were designed using the PyroMark assay design 2.0 software. PCR reaction (25μL) was performed according to manufacturer’s instructions (PyroMark PCR kit, Qiagen, Hilden, Germany); containing 50 ng bisulfite-treated DNA and 400nM of forward primer and biotin-labeled reverse primer. The primer sequences and PCR conditions are summarized in **S1 Table**. Amplification was carried out as follows: 95°C 15 min, then 45 cycles of 95°C 30 sec, annealing temperature for 30 sec (**S1 Table**), 72°C for 30 sec, followed by 72°C for 10 min. Biotin-labeled PCR products were captured with Streptavidin Sepharose beads (GE Healthcare, UK), and made single stranded using a Pyrosequencing Vacuum Prep Tool (Qiagen, Hilden, Germany). Sequencing primer (**S1 Table**) was annealed to the single-stranded PCR product by heating to 80°C, followed by slow cooling. Pyrosequencing was performed on a PyroMark Q24 system (Qiagen, Hilden, Germany) and cytosine methylation was quantified using PyroMark Q24 1.010 software.

### Statistical analysis

The normalized β-values of the 450K BeadChip data were converted to M-values (M = log2(β/(1-β)) and differential methylation between samples (blood vs. saliva as well as RA cases vs. healthy controls) was estimated with linear models using R-software package Limma (v3.20.9) [34,35]. Resulting *P*-values were corrected for multiple testing using the Benjamini-Hochberg procedure (P_Adj_). Results in tables and figures are presented as median β-values ± standard deviation.

Correlations between methylation in saliva and PBMC samples were measured using the Pearson’s correlation coefficient. A Benjamini-Hochberg corrected P-value <0.05 was considered as significant.

Differences in cell type compositions between RA cases and controls were compared using the student t-test. One-way ANOVA was used to find differences in methylation between cell types in the DMS between RA cases and controls.

The pyrosequencing data were analyzed with Mann-Whitney U tests to identify statistical differences between RA cases and healthy controls. Correlations between differentially methylated markers in PBMC vs. saliva, or RA cases vs. healthy controls were studied by calculating the Spearman rank correlation coefficients (ρ).

### Ingenuity Pathway Analysis

Canonical pathway analysis and network analysis was performed in Ingenuity Pathway Analysis (IPA) (http://www.ingenuity.com/). We used the IPA network score to determine the significance and specificity of each generated network. Canonical pathway analysis displays the most significant Canonical Pathways across the entire dataset. The significance values were calculated using the Fisher's exact test. A -log (p-value) cutoff of 1.3 (P<0.05) was applied. For each pathway also the ratio indicating the number of genes in a particular pathway over the total number of genes that make up the pathway according to IPA Knowledge base was calculated.

## Results

### Characteristics of the study population

Non-smoking subjects were enrolled in this study. The average age was 37.2 years ± 6.1; 36.8 ± 5.1 years for the control group and 37.6 ± 7.6 years for the RA group. There were 2 and 3 female participants in the RA and control groups, respectively. The RA cases were mainly allergic for house dust mite (4 out of 5 cases) or suffered from hay fever (3 out of 5 cases), while the prevalence of asthma and rhinitis was slightly lower (only 2 out of 5 cases). Preventive medicine (i.e. inhalation of a combination of a β2-agonist and corticosteroid) was taken daily by 2 of the RA cases, while 2 other RA cases only used medicine (either a combination of β2-agonist/anticholinergic inhaler with steroids nose spray and antiallergic eye drops, or oral intake of antihistamines combined with a steroid inhaler) when suffering from an upsurge. None of the healthy controls reported family allergy, while 2 of the 5 RA cases stated one or both of their parents to suffer from RA.

### Comparison of blood and saliva DNA methylation patterns

**Fig 1:**
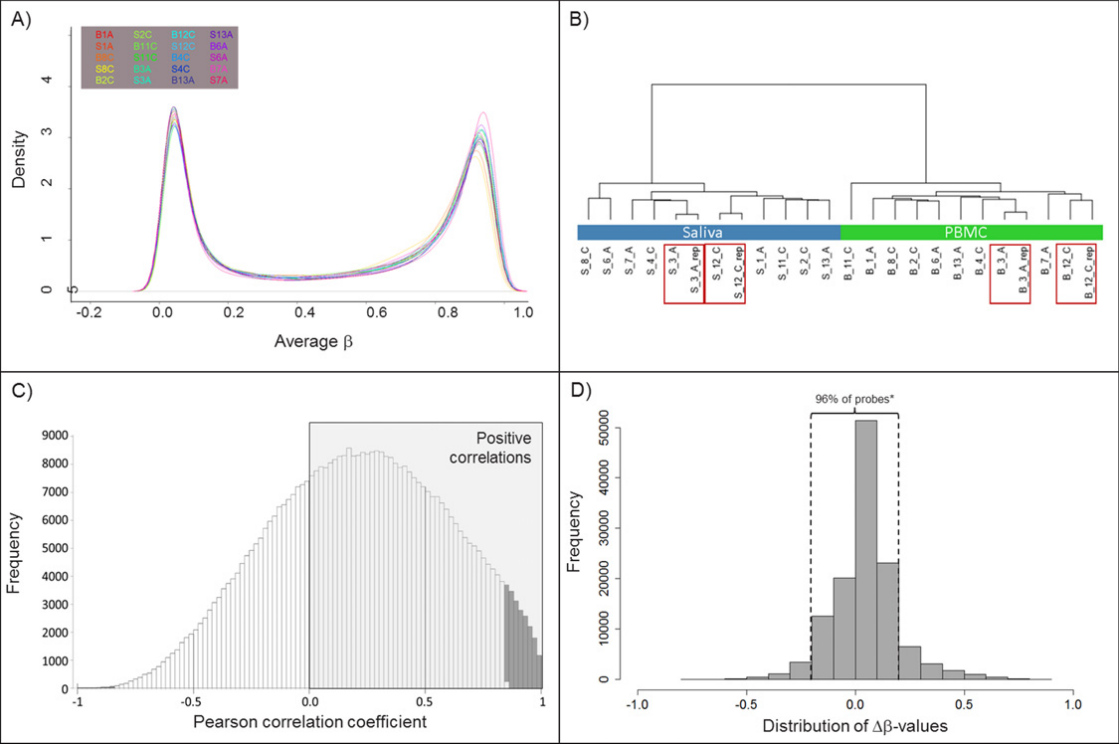
Data clustering and comparison between PBMC and saliva DNA methylation profiles. A) Density plots for methylation levels (expressed as average β-values) from PBMC and saliva samples from all 10 individuals; B) Hierarchical clustering based on correlation distances of whole genome methylation profiles in 24 samples; including PBMC and saliva samples from 10 individuals + technical replicates of PBMC and saliva for 2 individuals (red boxes); C) Pearson correlation coefficients for methylation levels of PBMC versus saliva for each of the CpG sites (positive correlations in grey area, statistically significant correlations (P_adj_<0.05) are shown as grey bars); D) The number of cg-probes that showed significant differential methylation in saliva versus PBMC (P_adj._<0.001), plotted against the difference in methylation expressed as Δβ-values. * The majority of the CpG sites (96%) show less than 20% difference in methylation between blood and saliva. *Samples are coded with either B for PBMC or S for saliva*, *followed by the ID number and C for control or A for RA cases group; _rep indicates technical replicates*.

Illumina 450K data were generated for 24 samples (saliva and PBMC from 10 individuals), with four samples in duplicate to evaluate the technical reproducibility. Approximately 7% (N = 33,846) of cg-probes were removed according to the quality control criteria mentioned in the materials and methods section. Following quality filtering and normalization 451,731 individual cg-probes were included in downstream data analyses. Density plots showed a typical pattern for each of the samples and highly comparable distributions indicating success with the BMIQ normalization procedure (**Fig 1A**). Hierarchical clustering based on correlation distances showed that PBMC and saliva samples separated into two clusters and technical replicates clustered together (**Fig 1B**). For 69.8% of CpG sites positive Pearson correlations between methylation levels of PBMC and saliva were observed (**Fig 1C**), indicating that the majority of DMS showed the same polarity of methylation levels in saliva versus PBMC, though only 4.6% of the correlations were statistically significant (P_Adj_<0.05). According to this criterion, 96% of the CpG sites show a similar DNA methylation pattern between PBMC and saliva. In **Fig 1D** we show the distribution of difference in DNA methylation (|Δβ|) between PBMC and saliva. Using the filtering procedure of P_Adj_<0.001 and ≥20% |Δβ|, we identified 17,984 differentially methylated sites, of which 5,168 (29%) CpG sites hyper- and 12,813 (71%) hypomethylated in saliva DNA compared to PBMC.

The list with 17,984 DMS between saliva and PBMC was reduced to 13,757 DMS by considering only those that could be mapped to annotated genes. Probes mapped in the same gene were removed and this resulted in a list of 6,246 unique genes for the IPA analysis. When looking at the top 5 canonical pathways (**Table 1**), differentially methylated genes were mainly involved in pathways responsible for cell signaling, some of which were involved in the regulation of immune responses. The IPA top networks were mostly linked with organismal injury and abnormalities, cancer and hereditary disorder. The full IPA summary report has been provided as supplementary material (**S1 Report**).

**Table 1:** The top 5 canonical pathways that show differential methylation between PBMC and saliva.

Ingenuity Canonical Pathways	-log(p-value)	Ratio
Molecular Mechanisms of Cancer	2.06E01	187/365
Integrin Signalling	1.12E01	102/201
Tec Kinase Signalling	1.12E01	85/158
T Cell Receptor Signalling	1.09E01	59/97
Leukocyte Extravasation Signalling	1.08E01	100/198

Since saliva and PBMC samples are a heterogeneous collection of cell types, each with a very different DNA methylation profile, we applied a statistical deconvolution method that permits estimating the relative proportion of cell types from DNA methylation profiles (**S1 and S2 Figs**). As expected the PBMC samples consisted of lymphocytes (mainly CD4^+^-T cells, NK cells and B cells) and monocytes. Also saliva contained mainly mononuclear cells, especially CD4^+^-T cells and monocytes, but there was also a reasonable proportion of buccal cells.

### Common DMS in blood and saliva associated with RA

**Fig 2:**
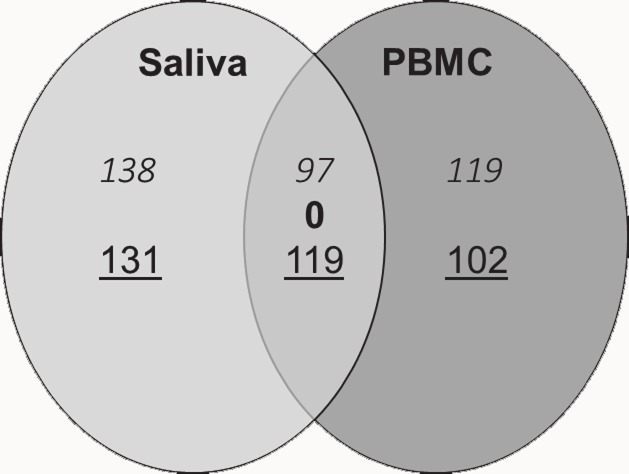
Identification of differentially methylated probes between RA cases and healthy controls (485 DMS in saliva and 437 DMS in PBMC). 216 probes were in common in PBMC and saliva. The common probes showed the same polarity of methylation levels (*hypermethylated*, **contramethylated**, hypomethylated).

**Fig 3:**
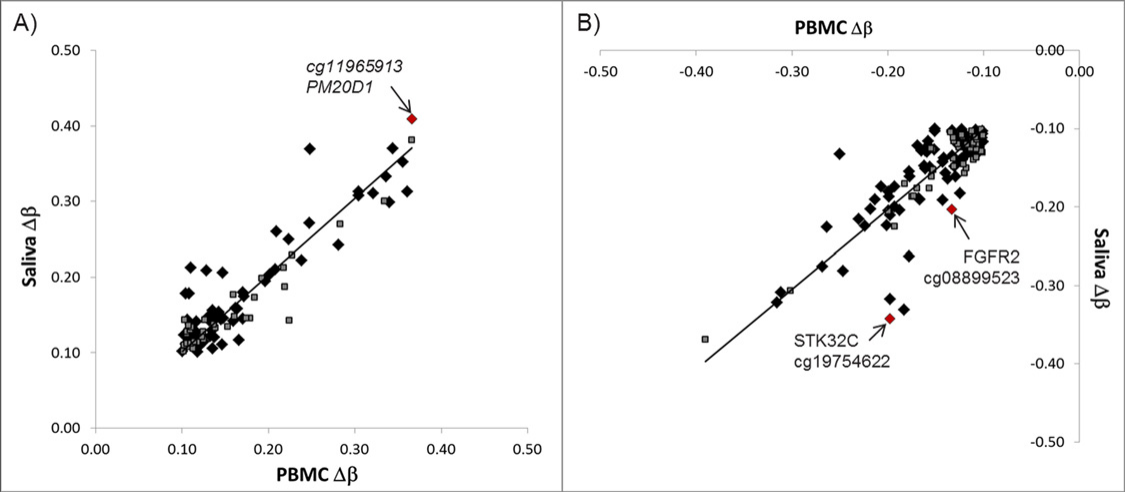
Correlation for hypermethylated (A; ρ = 0.78, P<0.001) and hypomethylated (B; ρ = 0.74, P<0.001) probes in common between PBMC and saliva. Black diamonds represent DMS located in annotated genes; grey squares are non-annotated DMS; red diamonds correspond to the CpG sites in 3 different genes that were selected for verification via bisulfite pyrosequencing.

When adopting a P_adj_<0.05, only 2 CpG sites were identified to be differentially methylated between RA cases and controls in saliva and only 1 site in blood; of which cg06751007 in *RNF213* was in common between saliva and blood (Δβ = -18.7 and -19.9, respectively). In the discovery phase we further defined differential methylation as those probes having at least 10% difference in DNA methylation level (Δβ<-0.1 or >0.1) and passing the threshold of a non-adjusted P<0.05. This approach increased the chance for identifying candidate sites that are differentially methylated and are relevant for respiratory allergy. We identified 485 CpG-sites in saliva and 437 CpG sites in PBMC that were differentially methylated between RA cases and controls with this double filtering procedure. The full list of DMS for saliva is given in supplementary **S2 Table**. Of these DMS, 216 CpG sites were in common between blood and saliva; 97 sites were hypermethylated and 119 sites were hypomethylated in the cases as compared to the healthy controls in PBMC and saliva **(Fig 2; S3 Table)**. Correlations between the differential methylation levels in blood and saliva samples revealed significant positive correlations for both hyper- (**Fig 3A**; ρ = 0.78, P<0.001) and hypomethylated CpG sites (**Fig 3B**; ρ = 0.74, P<0.001).

**Fig 4:**
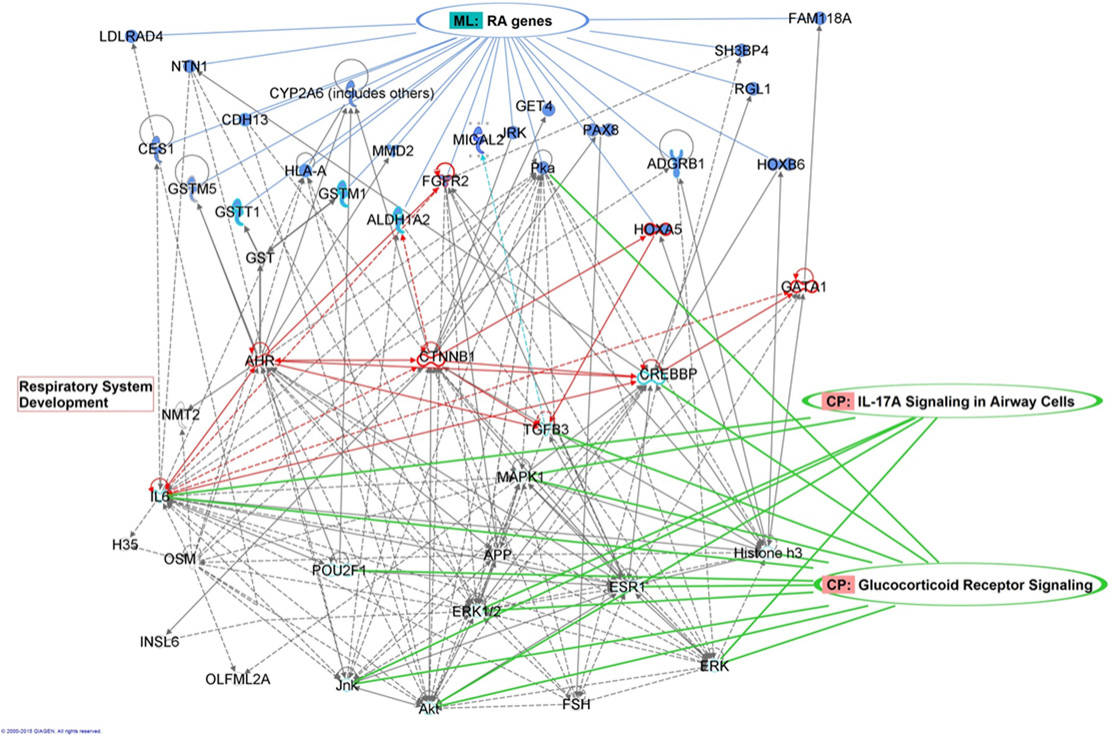
Network of genes involved in respiratory system development or inflammatory signaling. Twenty three of the focus genes are represented.

The 216 DMS between RA cases and controls are located in 85 unique annotated genes. The IPA Core Analysis of this 85-gene list indicated that canonical pathways and top disease and biofunctions were related to drug metabolism, xenobiotic detoxification and immune responses (**S2 Report**). The two top networks were related with drug metabolism and tissue development. A more detailed analysis was done by merging these networks and overlaying the combined network with the 85-gene list. Twenty three of the 85 focus genes were represented. The network was simplified by keeping only the nearest neighbors of the focus genes. Nine genes were involved in respiratory system development according to the IPA Knowledge Base, including *FGFR*, *HOXA5* and *ALDH1A2*. The network included key inflammatory molecules such as IL6 and kinases such as MAPK1, ERK1/2 and transcription factors (CREB) that are involved in glucocorticoid receptor signaling and IL17 signaling in airway cells (**Fig 4**).

**Fig 5:**
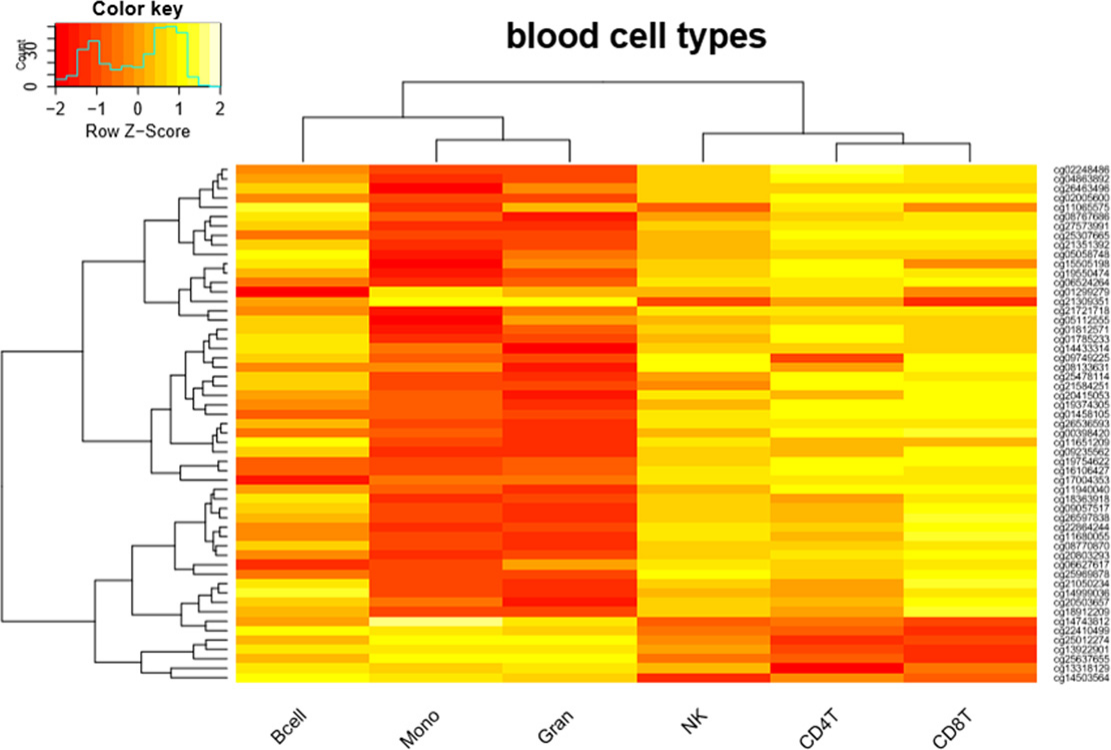
Differential methylation between blood cell types. Of the 216 DMS between allergy and control 55 sites are differently methylated between blood cell types (P<0.05).

Applying the Houseman statistical deconvolution method allowed us to evaluate whether allergy associated DNA methylation changes might be due to alterations in immune cell/epithelial buccal cell composition in the different samples. Of special note, 55 out of the 216 DMS between RA cases and controls were differentially methylated between the blood cell types in reference epigenomes (**Fig 5**). However, upon blood cell type specific deconvolution of DNA methylomes of RA cases and controls, no significant changes in relative immune cell/epithelial buccal cell distribution could be observed between PBMC and saliva samples (**S3 and S4 Figs**).

### Verification of 450K methylation data by pyrosequencing

To confirm the 450K BeadChip data, three CpG sites located in annotated genes that showed the most pronounced differences in DNA methylation between RA cases and controls in saliva were selected for confirmation by pyrosequencing (red diamonds in **Fig 3; S3 Table**): 1) cg19754622 located in *STK32C* (-34% hypomethylated), 2) cg11965913 located in *PM20D1* (41% hypermethylated), and 3) cg08899523 located in *FGFR2* (hypomethylation of -20%). Differences in DNA methylation levels between RA cases and controls for the 3 selected CpG sites were of similar magnitude as observed with the 450K BeadChips, though, differences were only statistically significant for saliva (**Table 2**). Significant correlations between the pyrosequencing and 450K BeadChip data were found; ρ ranged from 1.00–0.85, P≤0.002. Differential methylation levels of the 3 selected CpG sites detected in PBMC were significantly correlated with levels detected in saliva for both assays (*STK32C*: ρ_pyroseq_ = 0.76, P_pyroseq_ = 0.011, ρ_array_ = 0.73, P_array_ = 0.016; *FGFR2*: ρ_pyroseq_ = 0.82, P_pyroseq_ = 0.004; and *PM20D1*: ρ_pyroseq._ = 0.98, P_pyroseq_<0.001, ρ_array_ = 0.94, P_array_<0.001), apart from *FGFR2* levels detected via array (ρ_array_ = 0.59, P_array_ = 0.074 PBMC vs. saliva).

**Table 2:** Comparison of methylation differences between RA cases and controls in PBMC and saliva as assessed by Illumina 450K bead chips and bisulfite pyrosequencing.

Assay	cg-probe	gene	Blood PBMC			Saliva		
			Mean β (%) ±SD		Δβ (%)	Mean β (%) ±SD		Δβ (%)
			RA cases	Controls		RA cases	Controls	
450K^1^	cg19754622	STK32C	52.39 ± 5.3	72.17 ± 21.0	-19.78*	30.62 ± 13.0	64.82 ± 28.5	-34.21*
	cg08899523	FGFR2	45.15 ± 10.5	58.47 ± 9.5	-13.32*	39.52 ± 15.2	59.81 ± 9.5	-20.30*
	cg11965913	PM20D1	55.24 ± 36.6	18.65 ± 17.6	36.58*	62.63 ± 33.7	21.69 ± 20.4	40.94*
pyroseq.^2^	cg19754622	STK32C	43.10 ± 8.8	64.20 ± 19.4	-21.10	20.07 ± 11.4	51.30 ± 23.1	-31.23*
	cg08899523	FGFR2	26.47 ± 8.5	36.87 ± 9.4	-10.40	24.60 ± 12.6	39.33 ± 9.6	-14.73^
	cg11965913	PM20D1	60.47 ± 32.5	26.00 ± 21.8	34.47	64.20 ± 32.9	27.47 ± 19.9	36.73*

Statistics were performed

^1^using M-values with Limma package, or

^2^Mann-Witney U test to study differences between RA cases and controls (*P≤0.05; ^P<0.1).

## Discussion

We report the results of a pilot study that addresses the applicability of whole-methylome profiling in saliva in comparison to the corresponding pattern obtained from blood mononuclear cells of the same individuals. We recruited individuals with doctors’ diagnosed RA and healthy controls in order to identify differential methylation in blood or saliva that may relate to the clinical condition. Saliva yielded good quality DNA that passed as efficient as DNA from blood cells the different steps in the Illumina Methylation 450K BeadChip protocol. Saliva and blood methylation patterns were clearly distinguishable though the methylation status of about 96% of the CpG sites was comparable between PBMC and saliva. When comparing RA cases with healthy controls, the number of DMS in saliva and PBMC were 485 and 437, respectively, of which 216 were in common and showing the same polarity between blood and saliva. Pyrosequencing analysis of 3 selected DMS confirmed the array data.

### Saliva as alternative biofluid for DNA methylation profiling

Saliva can be collected more easily and with fewer constraints than blood samples. Similarity between the methylation patterns of the two biofluids makes saliva attractive as a source of DNA for epigenetic biomarker screening in various disease models. Only a few studies have recently addressed the question about the resemblance of the DNA methylation patterns in both biofluids [14–16], mainly focusing on assessing DNA methylation patterns in saliva vs. blood from healthy volunteers either using a genome-wide [14,15] or gene-targeted approach [16]. Moreover, DNA methylation patterns are cell specific and biomarker candidates found in different tissues may simply reflect variable proportion of each cell type in these tissues [36,37]. The presence of bacterial DNA in saliva has also been regarded as a limitation. However, DNA purified from the saliva DNA collection kit used in the current study has been shown to contain a low bacterial DNA content (median 11.8%), which is about 5–8 times lower as compared to DNA obtained from mouthwashes (median bacterial DNA content 60%) or buccal swabs (median bacterial DNA content 90%) [38]. In genome-wide screening approaches bacterial sequences might compete with the human DNA in the hybridization steps, though, in our current study all samples passed quality controls and there were no indications bacterial DNA negatively influenced the microarray hybridization process. In addition, data were confirmed via gene-specific bisulfite pyrosequencing. This analysis is less subject to the presence of remaining bacterial DNA because of the gene-specific amplification of human DNA. Results from Illumina arrays and pyrosequencing were highly comparable and this indicates that presence of bacterial DNA was probably not a major issue in our study.

We compared our data to the observations done by Thompson and colleagues [15]. The latter authors generated genome-wide DNA methylation profiles of whole blood and saliva samples of healthy adults on an Illumina 27K platform, observing 1.8% of the probes to be differentially methylated. Whole blood consists of cells that have their own epigenetic profile and, therefore inter-individual cellular heterogeneity may influence the outcome of epigenetic studies [39]. Recently, Adalsteinsson et al. compared methylation levels for multiple CpGs in whole blood and PBMC. They concluded that the different cell types present in whole blood may have an influence on DNA methylation measurements and that the use of PBMC pellets, containing mainly monocytes and lymphocytes, reduces confounding effects [39]. In our study, after blood cell/epithelial buccal cell type specific deconvolution of DNA methylomes of RA cases and healthy controls, we did not observe significant shifts in immune cell/epithelial buccal cell populations in PBMC blood and saliva samples. Studies report that varying cell composition may explain apparent age-associated differences [40] or affect differential methylation associated with inflammatory diseases [32]. Cell type heterogeneity and changes in this heterogeneity because of external or internal factors may affect genome-wide results. We estimated this heterogeneity a posteriori by applying the Houseman deconvolution method, but did not find differences between our cases and controls. We guess that the study may have been too small to answer this complex question. For future studies, we propose to determine cell populations in blood and saliva aliquots and use this information in the statistical analysis. Alternatively, epigenomic profiling of specific immune cell subpopulations may be recommended to identify robust epigenetic biomarkers in RA subjects.

Next we compared saliva versus PBMC and observed 4% of the cg-probes on the 450K bead chip to be differentially methylated (*P*_Adj_<0.001 and |Δβ|>0.2). Using the same cut offs for DMS as Thompson et al. [15]; in particular P_adj_< 0.001 and Diff.score>|30|, as well as selecting probes present in the 27K platform only, almost an identical number of DMS between PBMC and saliva (1.8% when only considering 27K probes) could be identified with our current data. This means that the majority of probes were similarly methylated in PBMCs and saliva.

### Applicability of salivary epigenomics in the field of RA

We identified 216 DMS (corresponding to 85 unique genes) between RA cases and healthy controls that were in common between blood and saliva. Pathway analysis of this list of genes showed that genes were linked to drug metabolism and xenobiotic detoxification, and to a minor extent associated with immune responses such as the antigen presenting pathway (**S2 and S3 Tables**). The genes that were differentially methylated and that were in common between both biofluids showed consistent polarity of methylation levels and a good correlation between Δβ-values. We are not aware of any study which analyzed blood and saliva methylation patterns to differentiate between RA cases and healthy controls. In a different application field, Smith et al. [14] compared saliva and blood methylome with methylation patterns in different brain tissues. DNA methylation in saliva appeared more similar to patterns examined in cells from each of the brain regions than methylation in blood. The authors suggested that DNA methylation of saliva may offer distinct opportunities for epidemiological and longitudinal studies of psychiatric traits. Likewise, we also suggest that salivary DNA methylation profiles may offer distinct opportunities for molecular epidemiology studies of RA.

Three DMS located in *STK32C* (cg19754622), *FGFR2* (cg08899523) and *PM20D1* (cg11965913) were selected for confirmation via targeted pyrosequencing. The regions were selected based on the high methylation differences (|Δβ|≥0.2) observed between RA cases and controls in saliva. Significant correlations were observed between absolute methylation levels detected with array technology and bisulfite pyrosequencing. Significant methylation differences between RA cases and controls was only confirmed for *STK32C* (cg19754622) and *PM20D1* (cg11965913) in saliva using pyrosequencing. The fact that we failed to confirm the other DMS might be due to an interplay of the small size of the study in combination with the complexity of the problem studied (for example heterogeneity of RA patients and complex composition of the different biofluids).

The DMS mentioned above were in first instance selected as “proof of principle” (i.e. to confirm the array data), but the corresponding genes could be associated with respiratory and immunological responses. *STK32C* belongs to the protein kinase family and its function is still unclear. However, data mining of *STK32C* gene expression changes in Geoprofile datasets GSE26456 or GSE23014 revealed interesting correlations with CD4+ Treg-cell function and lung inflammation [41,42]. *FGFR2* is a fibroblast growth receptor involved in embryonic development and tissue repair [43]. Interestingly, using a candidate-gene approach in a group of 2,108 children and adolescents, a haplotype of SNPs in intron 17 of the *FGFR2* gene was found to be associated with atopy but not with airway hyperresponsiveness [44]. Hypomethylation of cg08899523 located in the *FGFR2* gene body was observed to be associated with RA in our study. Furthermore, FGFR2 plays a role in respiratory system development and interacts with molecules involved in IL17 signaling in airway cells according to the IPA Knowledge Base (**Fig 4**). At the *FGFR2* gene expression level, associations were identified with CD4+/CD8+ T-cell responses in severe asthma, dendritic cell response to Chlamydia pneumonia lung infection and PGE EP3 induced asthma (GEOprofile datasets GSE2276, GSE31773, GSE470, GSE12806) [45–47]. *PM20D1* codes for Peptidase M20 Domain Containing 1 and was found to be hypermethylated in association with obesity [48], stroke [49], childhood abuse [50] and now RA (current study). The only reference we found on methylation of *PM20D1* in association with RA, is a hypomethylation of the probe cg14893161 in infants born to mothers with asthma as well as atopic mothers without asthma [51]. Also, according to GEOprofile dataset GSE5112, *PM20D1* gene expression is regulated by the TLR5 pathway, which is critically involved in allergic responses and severe asthma [52–54].

In conclusion, our pilot study indicates that high quality methylation profiles can be generated from whole saliva using whole-methylome microarrays. Furthermore, we have compared differential methylation profiles in PBMC and saliva between individuals having RA and healthy controls. Our data shows that both biofluids share differentially methylated probes that are strongly correlated. Gene regions, such as those selected for validation of the method (*STK32C*, *FGFR2* and *PM20D1*) showed high methylation difference detected between the groups. We identified a total of 216 DMS that deserve further study, in bigger clinical cohorts, for their involvement in RA. We acknowledge the limitation of the size of our pilot study and are cautious to extrapolate our findings. The small study necessitated more relaxed statistical cutoff criteria and this may have led to false positives, an aspect that warrants further investigation. From an application point of view, we advocate the analysis of saliva that will simplify the assessment of DNA methylation patterns in molecular epidemiology studies, especially to increase compliance with vulnerable populations such as children. The use of different biofluids in epigenomics studies will aid in providing new levels of insight in the molecular mechanisms through which environmental factors and interventions can alter an individual’s risk of RA.

## Supporting Information

S1 Fig Contribution of various cell types in saliva samples. (PPTX)

S2 Fig Contribution of various cell types in PBMC samples. (PPTX)

S3 Fig Blood cell type specific deconvolution of DNA methylomes of RA cases versus controls for saliva samples. (PPTX)

S4 Fig Blood cell type specific deconvolution of DNA methylomes of RA cases versus controls for PBMC samples. (PPTX)

S1 Table Primer sequences and conditions for PCR reaction and pyrosequencing. (XLSX)

S2 Table DMS in saliva between RA cases and controls. (XLSX)

S3 Table DMS between RA cases and controls that are in common between PBMC and saliva (XLSX)

S1 Report IPA summary report for differentially methylated genes between saliva and PBMC. (PDF)

S2 Report IPA Core Analysis report for the 85 differentially methylated genes between RA cases and controls that were in common in saliva and PBMC. (PDF)
