# Correction: Whole-Genome Saliva and Blood DNA Methylation Profiling in Individuals with a Respiratory Allergy

**DOI:** 10.1371/journal.pone.0183088

**Published:** 2017-08-07

**Authors:** Sabine A. S. Langie, Katarzyna Szarc vel Szic, Ken Declerck, Sophie Traen, Gudrun Koppen, Guy Van Camp, Greet Schoeters, Wim Vanden Berghe, Patrick De Boever

There is a typographical error in Table 2. Table 2 reports averaged values of multiple CpG sites in the reported gene regions instead of the average of the single CpG site corresponding to the validated cg-probes. For instance, for cg19754622 in the STK32C gene region (S1 Dataset), the authors reported the average of the 6 CpG sites that were analyzed along with the CpG-site of interest (cg19754622 = CpG1). A similar switch of data for the other two gene regions is also present.

**Table 2:** Comparison of methylation differences between RA cases and controls in PBMC and saliva as assessed by Illumina 450K bead chips and bisulfite pyrosequencing.

Assay	cg-probe	gene	Blood PBMC			Saliva		
			Mean β (%) ±SD		Δβ (%)	Mean β (%) ±SD		Δβ (%)
			RA cases	Controls		RA cases	Controls	
450K^1^	cg19754622	STK32C	52.39 ± 5.3	72.17 ± 21.0	-19.78*	30.62 ± 13.0	64.82 ± 28.5	-34.21*
	cg08899523	FGFR2	45.15 ± 10.5	58.47 ± 9.5	-13.32*	39.52 ± 15.2	59.81 ± 9.5	-20.30*
	cg11965913	PM20D1	55.24 ± 36.6	18.65 ± 17.6	36.58*	62.63 ± 33.7	21.69 ± 20.4	40.94*
pyroseq.^2^	cg19754622	STK32C	52.00 ± 9.8	72.00 ± 18.6	-20.00	25.20 ± 14.6	61.20 ± 27.8	-36.00*
	cg08899523	FGFR2	39.80 ± 9.0	53.27 ± 11.4	-13.47	31.20 ± 14.9	51.60 ± 13.2	-20.40^
	cg11965913	PM20D1	59.00 ± 32.6	25.20 ± 24.3	33.80	64.70 ± 33.9	24.80 ± 21.5	39.90*

Statistics were performed: ^1^using M-values with Limma package, or ^2^Mann-Witney U test to study differences between RA cases and controls (*P≤0.05; ^P<0.1).

The statistical analyses were performed with the correct values, so this error does not affect the results or conclusions of this study. Please see the corrected Table 2 here.

## Supporting information

S1 Dataset Pyrosequencing Data. (XLSX)
