# Supplementary figures and images for: Whole-Genome Saliva and Blood DNA Methylation Profiling in Individuals with a Respiratory Allergy
Source: PLoS One. 2016 Mar 21;11(3):e0151109. doi: 10.1371/journal.pone.0151109 (PMC4801358; doi:10.1371/journal.pone.0151109)

## Slide 1
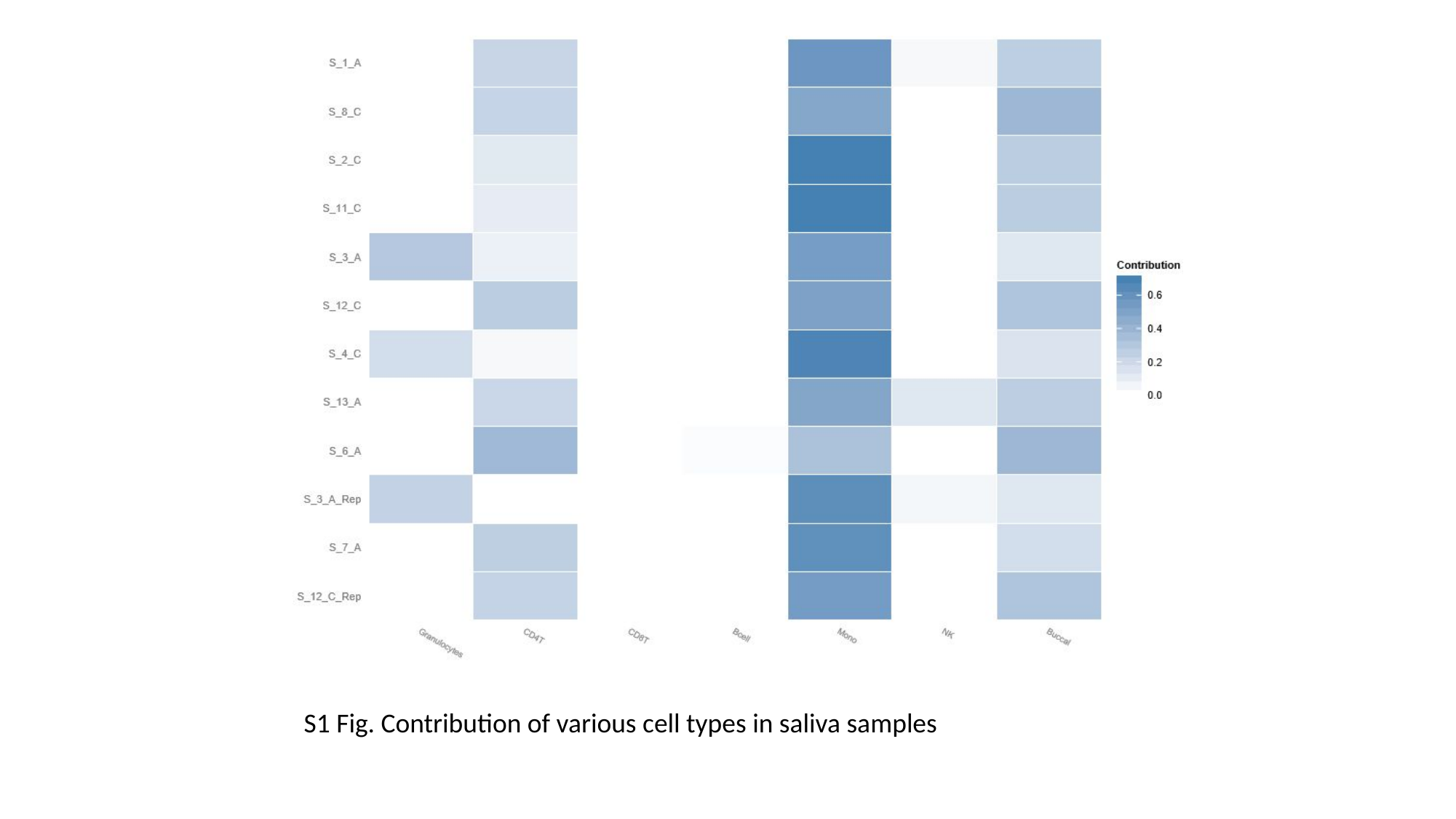

# S1 Fig. Contribution of various cell types in saliva samples

Supplement: S1 Fig — (PPTX) [file pone.0151109.s001.pptx]

## Slide 1
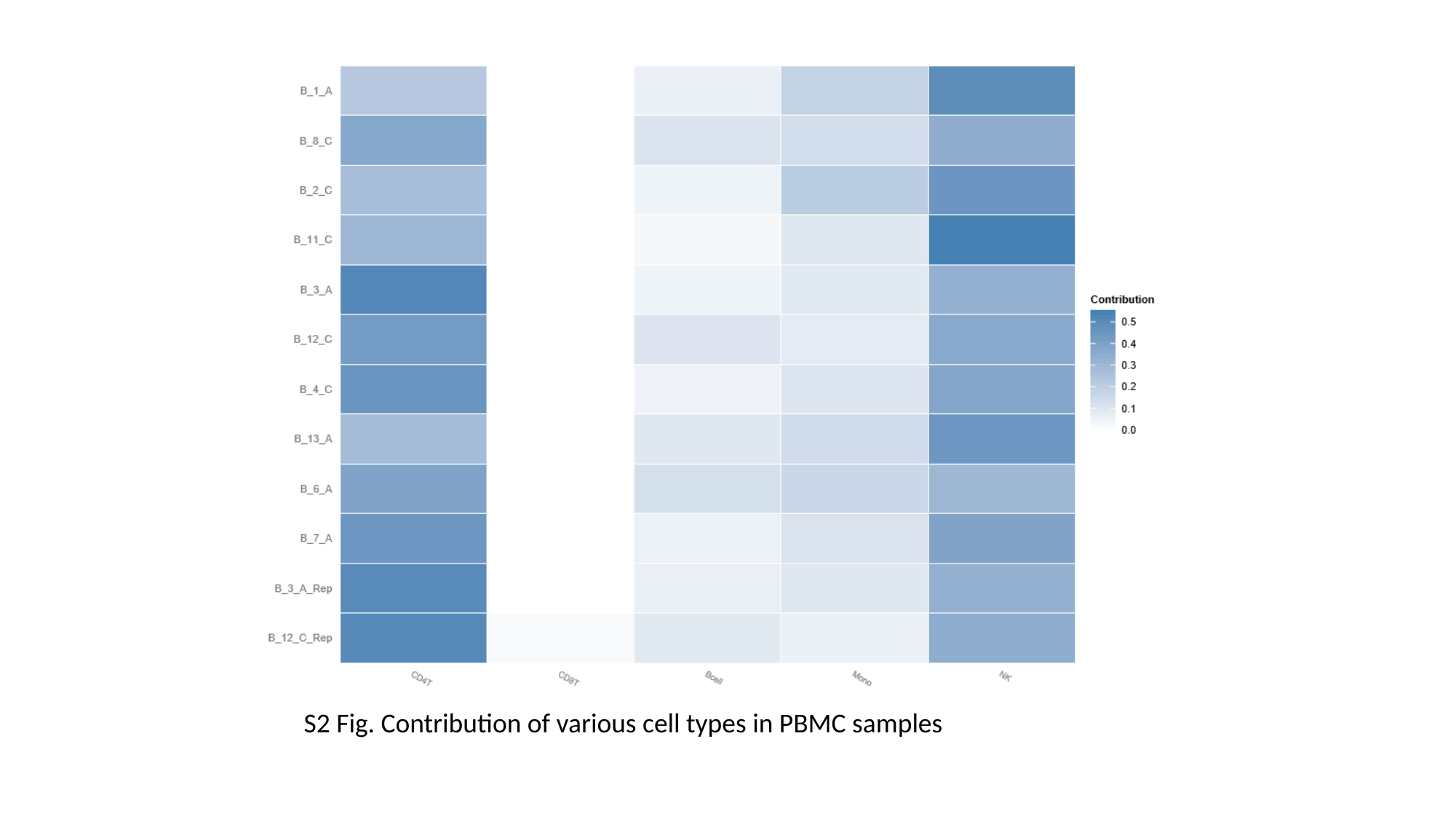

S2 Fig. Contribution of various cell types in PBMC samples

Supplement: S2 Fig — (PPTX) [file pone.0151109.s002.pptx]
