# Supplementary material for: Whole-Genome Saliva and Blood DNA Methylation Profiling in Individuals with a Respiratory Allergy
Source: PLoS One. 2016 Mar 21;11(3):e0151109. doi: 10.1371/journal.pone.0151109 (PMC4801358; doi:10.1371/journal.pone.0151109)
Supplement: S4 Fig — (PPTX) [file pone.0151109.s004.pptx]

## Slide 1
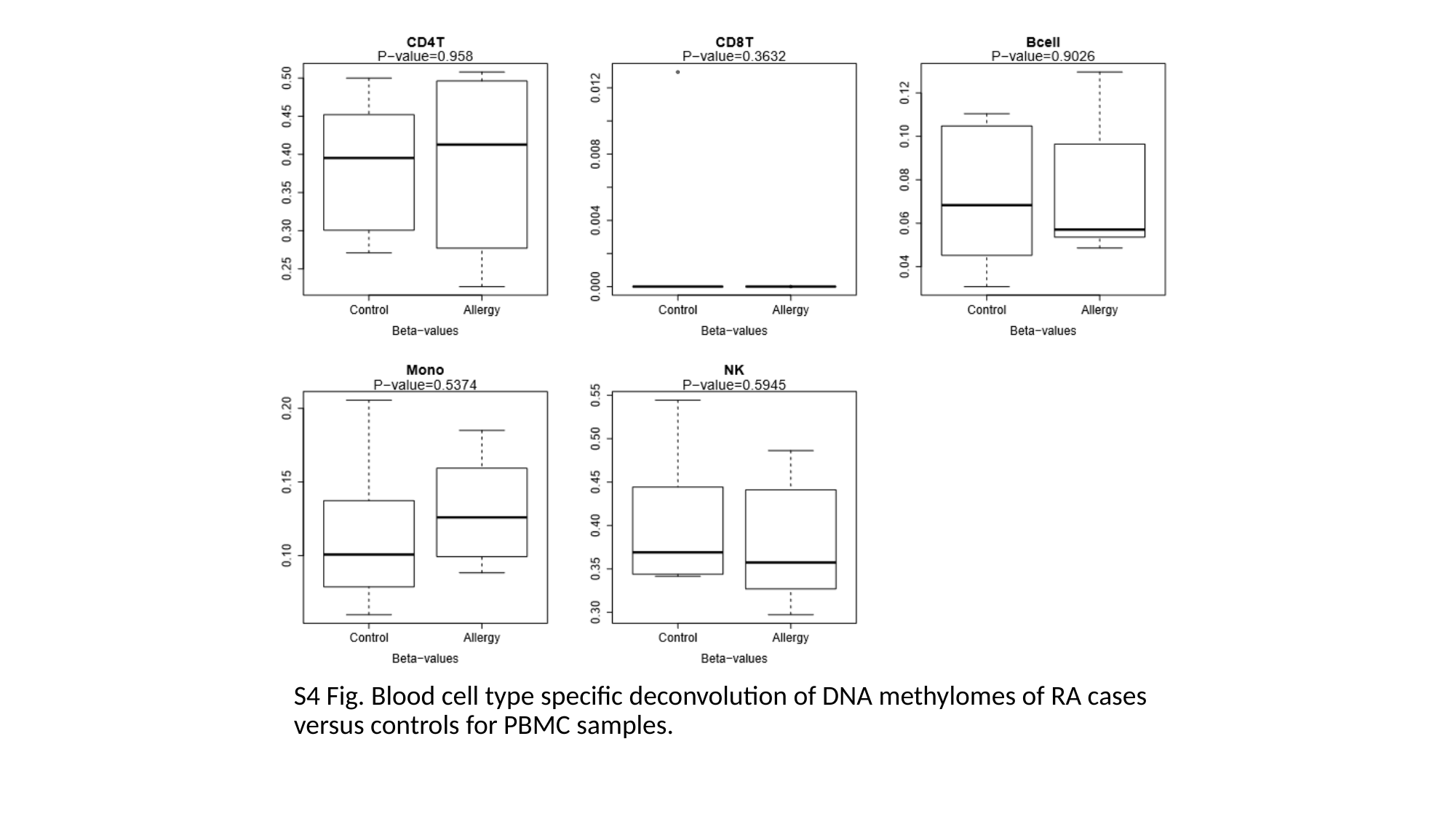

S4 Fig. Blood cell type specific deconvolution of DNA methylomes of RA cases versus controls for PBMC samples.
