# Supplementary material for: Whole-Genome Saliva and Blood DNA Methylation Profiling in Individuals with a Respiratory Allergy
Source: PLoS One. 2016 Mar 21;11(3):e0151109. doi: 10.1371/journal.pone.0151109 (PMC4801358; doi:10.1371/journal.pone.0151109)
Supplement: S1 Report — (PDF) [file pone.0151109.s008.pdf]

INGENUITY
PATHWAY ANALYSIS

Analysis Name: list of genes blood vs saliva - 2015-08-05 03:56 PM
Analysis Creation Date: 2015-08-05
Build version: 346717M
Content version: 24390178 (Release Date: 2015-06-17)

Analysis Settings

Reference set: Ingenuity Knowledge Base (Genes Only)

Relationship to include: Direct and Indirect

Does not Include Endogenous Chemicals

Optional Analyses: My Pathways My List

Filter Summary:

Consider only relationships where

data sources = Ingenuity Expert Findings OR Ingenuity ExpertAssist Findings

(c) 2000-2015 QIAGEN. All rights reserved.

INGENUITY SYSTEMS

Top Canonical Pathways

Name	p-value	Overlap	
Molecular Mechanisms of Cancer	2.71E-21	51.2 %	187/365
Integrin Signaling	6.24E-12	50.7 %	102/201
Tec Kinase Signaling	6.62E-12	53.8 %	85/158
T Cell Receptor Signaling	1.36E-11	60.8 %	59/97
Leukocyte Extravasation Signaling	1.44E-11	50.5 %	100/198

Top Upstream Regulators

Upstream Regulator	p-value of overlap	Predicted Activation
TNF	1.17E-53	
TGFB1	3.24E-47	
lipopolysaccharide	9.10E-42	
TP53	4.22E-38	
camptothecin	3.46E-35	

Top Diseases and Bio Functions

Diseases and Disorders

Name	p-value	#Molecules
Cancer	8.11E-11 - 3.20E-46	2040
Organismal Injury and Abnormalities	8.11E-11 - 3.20E-46	2011
Inflammatory Response	7.88E-11 - 1.07E-38	1058
Immunological Disease	6.02E-11 - 1.83E-36	938
Connective Tissue Disorders	5.12E-11 - 1.74E-34	540

Molecular and Cellular Functions

(c) 2000-2015 QIAGEN. All rights reserved.

INGENUITY® SYSTEMS

Name	p-value	#Molecules
Cellular Movement	7.88E-11 - 8.31E-80	1223
Cellular Growth and Proliferation	5.29E-11 - 4.94E-77	1848
Cell Death and Survival	8.11E-11 - 1.35E-65	1739
Cellular Development	6.24E-11 - 2.39E-57	1715
Cellular Function and Maintenance	6.24E-11 - 4.58E-49	1145

Physiological System Development and Function

Name	p-value	#Molecules
Immune Cell Trafficking	7.88E-11 - 3.94E-55	723
Hematological System Development and Function	7.88E-11 - 2.86E-49	1095
Tissue Morphology	9.16E-12 - 7.04E-47	764
Hematopoiesis	6.28E-11 - 2.45E-41	551
Lymphoid Tissue Structure and Development	2.70E-11 - 2.49E-41	414

Top Tox Functions

Assays: Clinical Chemistry and Hematology

Name	p-value	#Molecules
Increased Levels of Alkaline Phosphatase	6.26E-01 - 4.88E-04	34
Increased Levels of Red Blood Cells	1.75E-03 - 1.75E-03	14
Increased Levels of AST	5.35E-01 - 2.07E-02	9
Increased Levels of LDH	2.97E-01 - 3.36E-02	11
Increased Levels of Albumin	5.35E-01 - 4.36E-02	10

Cardiotoxicity

(c) 2000-2015 QIAGEN. All rights reserved.

Name	p-value	#Molecules
Cardiac Necrosis/Cell Death	6.26E-01 - 1.06E-10	119
Cardiac Hypertrophy	6.26E-01 - 1.58E-09	149
Cardiac Proliferation	4.81E-01 - 4.73E-06	44
Cardiac Fibrosis	6.26E-01 - 1.52E-04	66
Heart Failure	5.61E-01 - 1.07E-03	91

Hepatotoxicity

Name	p-value	#Molecules
Liver Proliferation	6.26E-01 - 3.19E-10	99
Liver Fibrosis	4.81E-01 - 7.49E-10	90
Liver Damage	6.26E-01 - 3.18E-09	138
Liver Steatosis	1.00E00 - 6.18E-07	93
Hepatocellular Carcinoma	2.80E-01 - 1.08E-06	188

Nephrotoxicity

Name	p-value	#Molecules
Renal Necrosis/Cell Death	4.30E-01 - 2.22E-18	220
Renal Damage	4.81E-01 - 4.28E-06	88
Renal Proliferation	6.26E-01 - 2.39E-05	102
Kidney Failure	4.74E-01 - 7.81E-05	75
Renal Dysfunction	2.19E-02 - 1.75E-03	14

Top Networks

ID	Associated Network Functions	Score
1	Molecular Transport, Small Molecule Biochemistry, Carbohydrate Metabolism	21

(c) 2000-2015 QIAGEN. All rights reserved.

2	Cell-To-Cell Signaling and Interaction, Cellular Assembly and Organization, Nervous System Development and Function	21
3	Infectious Diseases, Nutritional Disease, Carbohydrate Metabolism	21
4	Amino Acid Metabolism, Small Molecule Biochemistry, Cell-To-Cell Signaling and Interaction	19
5	Cellular Development, Nervous System Development and Function, Cardiovascular System Development and Function	19

Top Tox Lists

Name	p-value	Overlap	
Renal Necrosis/Cell Death	4.57E-18	46.0 %	228/496
Cardiac Hypertrophy	2.78E-13	44.9 %	179/399
Cardiac Necrosis/Cell Death	4.79E-11	46.6 %	125/268
Liver Proliferation	9.53E-11	48.0 %	109/227
Increases Liver Damage	1.88E-09	55.7 %	59/106

Top My Lists

Name	p-value	Overlap	
Arylhydrocarbonreceptor	2.54E-04	42.2 %	57/135
Androgen	3.04E-04	43.6 %	48/110
up_cluster cell activation	1.05E-01	41.7 %	10/24
Cell activation	3.07E-01	42.9 %	3/7
New My List 1	3.13E-01	50.0 %	2/4

Top My Pathways

Name	p-value	Overlap	
Thesis Merelb	1.17E-03	54.5 %	18/33
Thesis Merel	5.92E-03	57.9 %	11/19
NRF2-mediated Oxidative Stress Response_2013	6.83E-03	36.7 %	65/177

(c) 2000-2015 QIAGEN. All rights reserved.

INGENUITY SYSTEMS

RNALaterPath	1.17E-02	61.5 %	8/13
thesis M pathway brief	4.38E-02	53.8 %	7/13

(c) 2000-2015 QIAGEN. All rights reserved.
