# Supplementary material for: Whole-Genome Saliva and Blood DNA Methylation Profiling in Individuals with a Respiratory Allergy
Source: PLoS One. 2016 Mar 21;11(3):e0151109. doi: 10.1371/journal.pone.0151109 (PMC4801358; doi:10.1371/journal.pone.0151109)
Supplement: S2 Report — (PDF) [file pone.0151109.s009.pdf]

INGENUITY®
PATHWAY ANALYSIS

Analysis Name: list of genes RA vs C - 2015-08-07 11:12 AM
Analysis Creation Date: 2015-08-07
Build version: 346717M
Content version: 24390178 (Release Date: 2015-06-17)

Analysis Settings

Reference set: Ingenuity Knowledge Base (Genes Only)

Relationship to include: Direct and Indirect

Does not Include Endogenous Chemicals

Optional Analyses: My Pathways My List

Filter Summary:

Consider only relationships where

data sources = Ingenuity Expert Findings OR Ingenuity ExpertAssist Findings

(c) 2000-2015 QIAGEN. All rights reserved.

INGENUITY® SYSTEMS

Top Canonical Pathways

Name	p-value	Overlap
Glutathione-mediated Detoxification	1.87E-04	10.3 % 3/29
Neuroprotective Role of THOP1 in Alzheimer's Disease	4.90E-04	7.5 % 3/40
LPS/IL-1 Mediated Inhibition of RXR Function	1.57E-03	2.3 % 5/219
Aryl Hydrocarbon Receptor Signaling	2.10E-03	2.9 % 4/140
PXR/RXR Activation	2.21E-03	4.5 % 3/67

Top Upstream Regulators

Upstream Regulator	p-value of overlap	Predicted Activation
SF3B2	2.52E-04	
lead acetate	3.35E-04	
POR	4.42E-04	
CREB1	4.81E-04	
KMT2A	7.65E-04	

Top Diseases and Bio Functions

Diseases and Disorders

Name	p-value	#Molecules
Developmental Disorder	4.97E-02 - 1.45E-04	9
Endocrine System Disorders	1.53E-02 - 1.45E-04	3
Cancer	4.94E-02 - 1.99E-03	20
Gastrointestinal Disease	4.94E-02 - 1.99E-03	11
Organismal Injury and Abnormalities	4.97E-02 - 1.99E-03	26

Molecular and Cellular Functions

(c) 2000-2015 QIAGEN. All rights reserved.

INGENUITY SYSTEMS

Name	p-value	#Molecules
Drug Metabolism	4.53E-02 - 5.98E-05	6
Cell-To-Cell Signaling and Interaction	3.41E-02 - 2.93E-03	8
Cell Cycle	4.89E-02 - 3.85E-03	5
Cell Death and Survival	4.53E-02 - 3.85E-03	11
Cell Morphology	4.53E-02 - 3.85E-03	5

Physiological System Development and Function

Name	p-value	#Molecules
Embryonic Development	4.53E-02 - 1.68E-04	7
Organismal Development	4.91E-02 - 1.68E-04	12
Tissue Development	4.58E-02 - 1.68E-04	13
Cardiovascular System Development and Function	4.58E-02 - 1.05E-03	8
Connective Tissue Development and Function	4.89E-02 - 1.49E-03	7

Top Tox Functions

Assays: Clinical Chemistry and Hematology

Name	p-value	#Molecules
Decreased Levels of Albumin	4.89E-02 - 1.91E-02	2
Increased Levels of LDH	5.63E-02 - 5.63E-02	1

Cardiotoxicity

Name	p-value	#Molecules
Bradycardia	1.16E-01 - 1.16E-01	1
Cardiac Arrythmia	1.16E-01 - 1.16E-01	1
Pulmonary Hypertension	2.04E-01 - 2.04E-01	1
Cardiac Proliferation	2.13E-01 - 2.13E-01	1

(c) 2000-2015 QIAGEN. All rights reserved.

INGENUITY SYSTEMS

Cardiac Damage	2.49E-01 - 2.49E-01	1

Hepatotoxicity

Name	p-value	#Molecules
Glutathione Depletion In Liver	5.98E-05 - 5.98E-05	3
Liver Inflammation/Hepatitis	2.68E-01 - 3.85E-03	2
Liver Damage	2.29E-01 - 7.69E-03	2
Liver Cholestasis	3.41E-02 - 3.41E-02	1
Liver Fibrosis	3.44E-01 - 3.44E-01	1

Nephrotoxicity

Name	p-value	#Molecules
Renal Damage	5.99E-02 - 3.85E-03	4
Kidney Failure	4.72E-02 - 3.80E-02	3
Renal Tubule Injury	5.99E-02 - 5.00E-02	3
Renal Necrosis/Cell Death	1.00E00 - 8.14E-02	2
Renal Proliferation	1.72E-01 - 1.72E-01	2

Top Networks

ID	Associated Network Functions	Score
1	Drug Metabolism, Glutathione Depletion In Liver, Embryonic Development	53
2	Tissue Development, Cell Death and Survival, Cell Morphology	39
3	Drug Metabolism, Molecular Transport, Nucleic Acid Metabolism	17
4	Cellular Development, Cellular Growth and Proliferation, Cell Morphology	11
5	Developmental Disorder, Hereditary Disorder, Ophthalmic Disease	2

(c) 2000-2015 QIAGEN. All rights reserved.

INGENUITY® SYSTEMS

Top Tox Lists

Name	p-value	Overlap	
Glutathione Depletion - Phase II Reactions	5.98E-05	15.0 %	3/20
Xenobiotic Metabolism Signaling	1.88E-03	1.8 %	6/336
PXR/RXR Activation	2.21E-03	4.5 %	3/67
LPS/IL-1 Mediated Inhibition of RXR Function	2.85E-03	2.0 %	5/251
Aryl Hydrocarbon Receptor Signaling	3.48E-03	2.5 %	4/161

Top My Lists

Name	p-value	Overlap	
Arylhydrocarbonreceptor	1.84E-03	3.0 %	4/135
Androgen en estrogen	3.10E-01	1.0 %	1/96
Androgen	3.47E-01	0.9 %	1/110

Top My Pathways

Name	p-value	Overlap	
AhR indirect interaction	9.21E-05	13.0 %	3/23
PXR indirect interaction	9.43E-04	16.7 %	2/12
NRF2-mediated Oxidative Stress Response_2013	3.10E-02	1.7 %	3/177
CAR indirect interaction	4.16E-02	9.1 %	1/11

(c) 2000-2015 QIAGEN. All rights reserved.
